# Small Molecules as Modulators of Voltage-Gated Calcium Channels in Neurological Disorders: State of the Art and Perspectives

**DOI:** 10.3390/molecules27041312

**Published:** 2022-02-15

**Authors:** Stefano Lanzetti, Valentina Di Biase

**Affiliations:** Institute of Pharmacology, Department of Medical Statistics, Informatics and Health Economics, Medical University of Innsbruck, Peter-Mayr Strasse 1, A-6020 Innsbruck, Austria; stefano.lanzetti@i-med.ac.at

**Keywords:** voltage-gated calcium channels, small molecules, splice variants, Ca_V_1, Ca_V_2, Ca_V_3, seizure, autism spectrum disorders, anxiety, pain, PYT, Compound **8**, gabapentin, pregabalin

## Abstract

Voltage-gated calcium channels (VGCCs) are widely expressed in the brain, heart and vessels, smooth and skeletal muscle, as well as in endocrine cells. VGCCs mediate gene transcription, synaptic and neuronal structural plasticity, muscle contraction, the release of hormones and neurotransmitters, and membrane excitability. Therefore, it is not surprising that VGCC dysfunction results in severe pathologies, such as cardiovascular conditions, neurological and psychiatric disorders, altered glycemic levels, and abnormal smooth muscle tone. The latest research findings and clinical evidence increasingly show the critical role played by VGCCs in autism spectrum disorders, Parkinson’s disease, drug addiction, pain, and epilepsy. These findings outline the importance of developing selective calcium channel inhibitors and modulators to treat such prevailing conditions of the central nervous system. Several small molecules inhibiting calcium channels are currently used in clinical practice to successfully treat pain and cardiovascular conditions. However, the limited palette of molecules available and the emerging extent of VGCC pathophysiology require the development of additional drugs targeting these channels. Here, we provide an overview of the role of calcium channels in neurological disorders and discuss possible strategies to generate novel therapeutics.

## 1. Introduction

Ion channels are the molecular underpinnings of membrane permeability and excitability and are essentially involved in the function of every organ in the body. Over fifty inherited channelopathies are attributed to ion channel dysfunctions [1]. Currently, small molecules targeting ion channels represent 18% of the drugs approved by the Food and Drug Administration, highlighting the importance of ion channels in clinical pharmacology [2]. The aberrant expression of VGCCs, mutations in their amino acid sequence, and altered post-transcriptional regulation are associated with several brain disorders and comorbidities [3,4,5,6,7,8,9,10,11,12]. Small molecules directed at VGCCs available in medical practice include blockers, some of which lack the selectivity to channels’ isoforms and cause pronounced side effects. These inhibitors are not sufficient to treat VGCC-dependent diseases. Therefore, new molecules targeting VGCCs need to be identified and characterized on channel-mediated functions. In this manuscript, we discuss the impact of alternative splicing on channel drug sensitivity, the importance of restoring proper calcium current kinetics in dysfunctional channels, and the efficacy of selective blockers in the treatment of pain and seizure. Furthermore, preclinical findings show that several small molecules that are in use for specific conditions of VGCCs may be potential candidates for additional applications. We report some compounds exhibiting isoform selectivity or the ability to offset aberrant signaling pathways downstream of calcium channel mutants that are associated with diseases. Ideally, innovative molecules should selectively target only those channels involved in pathological processes, while sparing those participating in normal functions. To this end, understanding the physio-pathological regulation of VGCCs and the underlying molecular and cellular mechanisms is paramount.

## 2. Voltage-Gated Calcium Channels

VGCCs are pore-forming multisubunit complexes that allow calcium influx upon membrane depolarization and control a plethora of tissue-specific processes, including excitation-contraction coupling, neurotransmitter and hormone release, gene transcription, synaptic plasticity, membrane excitability, and cardiac and neuronal pacemaker activity [13]. In the 1980s and early 1990s, VGCCs were classified into L-, N-, P/Q-, and R-types, based on their pharmacological, voltage-dependence, and kinetic properties (Table 1) [13,14]. Long-lasting L-type calcium currents are endowed with a large single-channel conductance and sensitivity to dihydropyridine (DHP), phenylalkylamines, and benzothiazepines [15]. T-type calcium channels activate at low voltages, inactivate rapidly, deactivate slowly, and are characterized by a tiny single-channel conductance [13,16,17]. N-type currents were first recorded in dorsal root ganglion neurons; being non-L-type and non-T-type, they were designated as neuronal [16]. N-type calcium currents are inhibited by the snail ω-conotoxin GVIA and the related molecules [18,19]. P-type currents were initially recorded in Purkinje neurons. P-type currents are typically DHP- and ω-conotoxin GVIA-insensitive, and are inhibited by the spider ω-agatoxin IVA [20,21]. Q-type currents were originally identified in cerebellar granule neurons. The ω-agatoxin IVA also blocks Q-type calcium currents with a lower affinity than the P-type [22]. These ω-agatoxin IVA-sensitive calcium currents are commonly referred to as P/Q-type. Finally, additional R-type currents were recorded in cerebellar granule neurons and were found to be sensitive to the tarantula toxin SNX-482 [23]. However, SNX-482 was later shown to be rather unselective as it also inhibits potassium channels [24].

**Figure 1:**
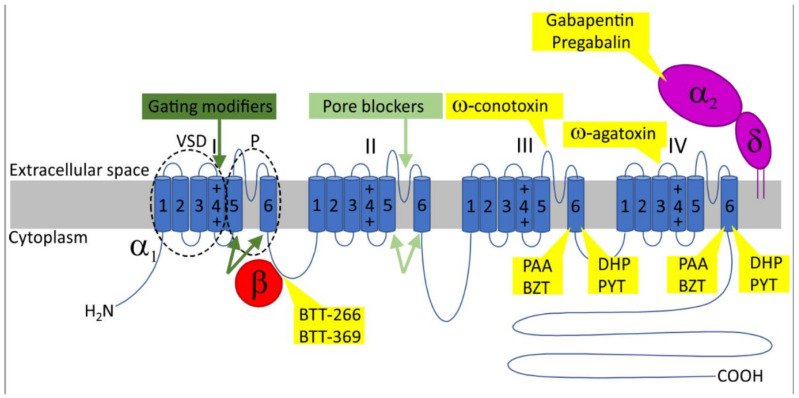
The topology of voltage-gated calcium channels with known drug-binding regions and the mechanisms of channel inhibition. The image represents the channel complex including the Ca_V_α_1_ pore forming subunit with the auxiliary Ca_V_β and Ca_V_α2δ which regulate channel trafficking and biophysical properties. The Ca_V_α_1_ is organized in four transmembrane domains (I–IV), each containing six membrane-spanning helices (S1–S6). All S5-S6 segments form the channel pore (P) whereas the S1-S4 constitute the voltage-sensing domain (VSD). Inhibition is achieved by modifying channel gating (dark green arrows, gating modifiers) through binding with the extracellular linkers of the VSD (e.g., agatoxin) or with the activation gates of the pore (e.g., DHP). Another blocking mechanism includes the direct occlusion of the pore from the extracellular space (e.g., conotoxin). Small molecules are membrane permeable and can access the pore from the cytoplasm, thereby impeding the ion permeation (light green, pore blockers) (e.g., PAA). BTT-266 and BTT-369 disrupt the Ca_V_α_1_–Ca_V_β interaction interfering with channel trafficking. Gabapentin and pregabalin reduce channel membrane expression by binding with the Ca_V_α2δ subunit. BZT, benzothiazepine; DHP, dihydropyridine; PAA, phenylalkylamine.

The advent of molecular cloning allowed the understanding of VGCCs at a molecular level and revealed the multi-subunit composition of the channel complex [13,75,76]. VGCCs consist of an α_1_ and associated β and α_2_δ subunits. The α_1_ constitutes the channel pore and allows calcium influx from the extracellular space into the cells, whereas the β and α_2_δ support channel trafficking and tune the kinetic properties of calcium currents (Figure 1). The α_1_ subunit comprises four homologous domains, each composed of six transmembrane helices. The four homologous domains are bridged via intracellular loops and are flanked by amino- and carboxy-terminal cytoplasmic regions, which function as a hub for multiple regulatory interactions and signaling mechanisms [13]. In 2000, a new nomenclature was adopted for VGCCs, grouping the α_1_ into Ca_V_1 (L-type), Ca_V_2 (non-L-type), and Ca_V_3 (T-type) [77]. The channel subunits are also termed Ca_V_α_1,_ Ca_V_β and Ca_V_α_2_δ by the scientific community in the field.

## 3. Physiological Roles of VGCCs in the Nervous System

VGCCs are ubiquitously expressed in the nervous system. Isoform diversity and localization confer specific functions to VGCCs [3,14,78,79,80,81,82,83,84]. L-type Ca_V_1.2 is the predominant L-VGCCs expressed in the brain and is highly represented in the cardiovascular system [80,85]. Ca_V_1.2 channels are localized on the soma and dendrites of most types of neurons, where they control gene expression [86,87,88], synaptic plasticity [89,90], calcium-dependent enzymes, and calcium-activated potassium channels [91]. Ca_V_1.3 channels exhibit a neuronal somatodendritic distribution that is similar to Ca_V_1.2. These two L-type isoforms are often co-expressed in the same neuronal type [85,92]. Ca_V_1.3 participates in postsynaptic signaling integration and regulates membrane excitability [3,44,93,94]. Ca_V_1.3 is also localized at the ribbon synapse of the inner cochlear hair cells, where it controls synaptic release and is necessary for the transmission of impulses to the auditory cortex [78,95,96]. Consistently, Ca_V_1.3 knock-out mice and humans expressing dysfunctional non-conducting Ca_V_1.3 mutants present with congenital deafness [42,43]. Ca_V_1.4 is predominantly expressed in the rod photoreceptors of the retina. Here, Ca_V_1.4 controls synaptic release and allows the transmission of visual stimuli [79]. Ca_V_1.4 knock-out mice are blind [97]. Mutations inducing loss-of-function in Ca_V_1.4 lead to night blindness in humans [47]. Ca_V_1.1 expression is restricted to the skeletal muscle, where it couples plasmalemma excitation with muscle contraction [5].

Ca_V_2.1 and Ca_V_2.2 channels localize at the presynapse of nerve cells [78]. Here, they integrate with the neurotransmitter release machinery by establishing interactions with local molecules [98,99]. Upon depolarization, calcium influx via Ca_V_2.1 and Ca_V_2.2 triggers the fusion of presynaptic vesicles with the membrane and, consequently, allows neurotransmitter release [98,100]. The relative content of Ca_V_2.1 and Ca_V_2.2 at the synapses may vary according to neuronal type [101]. For example, the synapses of the spinal dorsal horn express Ca_V_2.2 exclusively, whereas Ca_V_2.1 channels are also located in the soma of glutamatergic neurons. Here, Ca_V_2.1 mediates excitation-transcription coupling and has been associated with the ability to control the expression of the synaptic syntaxin-1A [87,102]. Gain-of-function mutations of Ca_V_2.1 cause familial hemiplegic migraine and impair synapse formation in animal models [49,103].

Ca_V_3 channels are expressed throughout the nervous system and are involved in cerebellar, thalamic, and cortical functions [104]. These channels are involved in the tuning of neuronal excitability and participate in the processing of pain, sleep, motor functions, and the release of neurotransmitters and hormones [11]. The three Ca_V_3 isoforms confer distinct firing patterns to neurons. A further level of regulation complexity is achieved by channel-alternative splicing [105,106]. A comprehensive recent review comprises the latest clinical findings on Ca_V_3 channelopathies and their underlying cellular mechanisms [11].

## 4. L-type VGCCs in Psychiatric Disorders

Genome-wide association studies identified an intronic single-nucleotide polymorphism, rs1006737, of the *CACNA1C* encoding the Cav1.2 channel as a risk factor for bipolar disorders [31,32], unipolar major depressive disorder [32,33,34], schizophrenia [33,35,36,37,38,39] and post-traumatic stress syndrome [40,41]. Individuals carrying the *CACNA1C* rs1006737 present altered neuronal circuitry in fMRI analysis, corroborating the central role of these channels in information processing in the brain [107]. In healthy humans, the *CACNA1C* rs1006737 is associated with decreased attention, working memory, and verbal fluency [38,107,108,109]. In line with these findings, the DHP isradipine was shown to improve verbal memory and attention in patients affected by schizophrenia in a recent randomized controlled trial [110]. How non-coding intronic single nucleotide polymorphisms can cause a pathological condition is unclear, but it is thought to happen by altering the expression level of Ca_V_1.2 and most likely the pattern of channel splice variants in the brain [111]. Recently, numerous splice variants of the human neuronal Ca_V_1.2 have been identified, and their sequences are available in an accessible repository [111]. Further research is needed to attribute specific functions to these splice variants in neurons. From a pharmacological point of view, alternative splicing may vary the sensitivity of L-type channels to DHP [112].

The *CACNA1D* gene encoding the Ca_V_1.3 has been identified as a risk gene for bipolar disorder [45,46]. In a pilot study on a very limited number of individuals with bipolar disorder, isradipine administration ameliorated the symptoms of comorbid depression [113]. Although it was too limited to draw valid conclusions, this study suggested a possible therapeutic application of L-type VGCCs antagonists in bipolar disorders. Therefore, this topic deserves further investigation. In this regard, a clinical trial (ClinicalTrials.gov identifier: NCT01784666) was approved but, unfortunately, it was prematurely terminated because of an insufficient enrollment of eligible patients.

Timothy syndrome (TS) is a multisystem disorder characterized by congenital heart disease, immunodeficiency, intermittent hypoglycemia, cognitive impairment, and autism [7,25]. This condition is associated with the mutually exclusive alternative splicing of the exons 8 and 8a of the Ca_V_1.2. In one form of TS, the pathogenic G406R mutation is located within the exon 8a. In a second form of TS, Ca_V_1.2 exhibits the G406R or a G402R mutation within the alternative spliced exon 8. Both forms of TS present autism, but the most pronounced traits are displayed in the TS associated with exon 8, as this splice variant is more expressed in the brain than the 8a [8,25]. The G406R substitution is a gain-of-function mutation and reduces voltage-dependent channel inactivation [26]. Interestingly, iPSC-derived neurons from individuals with TS showed an excessive expression of the tyrosine hydroxylase (*TH)* gene. Treatment with roscovitine—which increases channel inactivation [27,28] and, therefore, can rescue the inactivation impairment displayed by the G406R Ca_V_1.2 mutant—strongly reduced the redundant production of *TH*, restoring the physiological expression levels of this gene [88]. This finding is consistent with other studies in which roscovitine reduced the prolongation of the action potential in iPSC-derived cardiomyocytes from individuals with TS, reestablishing proper membrane excitability [29,114]. Interestingly, treatment with nifedipine did not retrieve adequate levels of *TH* production in TS-derived iPSCs neurons [29]. This result suggests that restoring the amplitude of calcium currents may not suffice to fully rescue an integrative physiological mechanism. Instead, restoring physiological channel kinetics is necessary. Consistently, the signaling mode of Ca_V_1.2 was previously reported to be either voltage- or calcium-dependent, suggesting the existence of multiple mechanisms by which the same channel can selectively control diverse cellular processes [115]. These data indicate that the tailored rational design of new molecules able to selectively target different gating modes can be fundamental to correcting the abnormal signaling pathways that are determined by channel mutations.

Several gain-of-function de novo missense mutations of Ca_V_1.3 are causative of the pathological conditions associated with intellectual disabilities, autism spectrum disorders, developmental delays, and hypotonia, as well as hyperinsulinemic hypoglycemia and/or congenital aldosteronism [44]. Interestingly, among all the described mutations, the germline Ca_V_1.3-S652L substitution shows increased sensitivity to isradipine [116], suggesting that the DHP-hypersensitivity of this channel mutant may be exploited for clinical practice. Therefore, further investigations in this direction are worthwhile. A recent review discusses in depth the Ca_V_1.3 gain-of-function mutations linked to autism and comorbidities, the underlying molecular mechanisms, clinical implications, and therapeutic potential of channel blockers [44]. Autism is also associated with single-nucleotide polymorphism in Ca_V_3 channel isoforms [117,118]. Furthermore, several missense mutations of the Ca_V_3.2 channel were identified in 6 out of 461 individuals with autism spectrum disorders. These mutations are located within channel domains that are highly conserved across species and were found to strongly reduce Ca_V_3.2 channel activity [73]. Such a loss of function may cause functional and structural alterations to the brain circuitry, leading to the development of autism [73]. A possible pharmacological treatment may include either promoting channel trafficking to the membrane or the administration of drugs able to increase Ca_V_3.2 activity.

Gabapentin and pregabalin (gabapentinoids) are effective in treating anxiety disorders in humans [119]. Because gabapentinoids target Ca_V_α_2_δ subunits, the anxiolytic efficacy of these compounds is consistent with the finding that Ca_V_α_2_δ1 level increased in a rat model in which anxiety was chemically induced [119]. Anxiety intimately connects with fear, and the underlying neural circuitries are tied [120]. In fear-conditioned rats, the expression of Ca_V_1.2 and Ca_V_1.3 was found to be upregulated, and the administration of nimodipine blocked the startle response in these rodents [121]. These results suggest that DHP could be used as an anxiolytic. Nevertheless, some discrepancy is found in additional studies. Ca_V_1.2 haploinsufficiency or its deletion in the forebrain were shown to induce an anxiety phenotype in mice [122]. Consistently, higher doses of nifedipine and verapamil exerted an anxiogenic effect in rodents [8,123]. Direct evidence that Ca_V_1.3 suppression may have an anxiolytic effect is weak [8,123]. Therefore, the role of Ca_V_1.2 in anxiety must be clarified to ponder the therapeutic potential of selective L-type channel blockers. Finally, Ca_V_2.2 knock-out mice show lower anxiety levels than wild-type mice, suggesting that inhibitors of Ca_V_2.2 might be potential anxiolytic drugs [124].

## 5. VGCC Inhibitors in the Treatment of Parkinson’s Disease

Parkinson‘s disease (PD) is a common neurodegenerative disorder, the incidence of which is progressively increasing. PD is characterized by a loss of dopaminergic neurons in the substantia nigra pars compacta and in the striatum. This neurodegeneration leads to a progressive impairment in motor skills, tremors, and development of psychosis [125,126]. The mainstay pharmacological treatment that is currently available targets the motor symptoms and includes several drugs as anticholinergic agents, beta-blockers, and dopamine receptor agonists [127]. Unfortunately, the etiology of loss in dopaminergic neurons is still unclear and this gap of knowledge strongly hampers tailored therapeutic interventions to avoid neurodegeneration. Research efforts provide an emerging frame comprising a network of contributing causes, including specific genes, environmental risk factors, and cellular metabolism stressors [128]. Multiple genes are involved in the development of PD, including α-synuclein, Parkin, PTEN-induced putative kinase 1 (PINK1), and leucine-rich repeat serine/threonine protein kinase 2 (LRRK2) [129]. Within the PD condition, these genes are often associated with mitochondrial dysfunction and calcium homeostasis dysregulation [130]. Interestingly, several epidemiological studies reported that the incidence of PD was reduced by 30% in patients treated with DHP for hypertension [131,132,133]. This observation suggested that L-type VGCCs are involved in the pathogenesis and/or progression of this neurological disorder and that DHP could be used to prevent the loss of neurons by inhibiting the L-type calcium channels. In line with this hypothesis, several other findings suggest that the upregulation of L-type Ca_V_1.3 may be critical in neuronal loss from PD [134]. In the substantia nigra dopamine neurons, Ca_V_1.3 contributes to pacemaker activity, which is sensitive to DHP [135,136]. Ca_V_1.3 pacemaker activity was shown to be linked to mitochondrial-dependent oxidative stress, which is typical of PD [137]. Furthermore, the Ca_V_1.3/Ca_V_1.2 expression ratio increases in favor of the Ca_V_1.3 in PD brains [138]. Altogether, these results indicate that selective inhibitors of Ca_V_1.3 channels could be a potential strategy for treating PD. However, selective Ca_V_1.3 inhibitors are not available in clinical practice, and the only possibility to test this hypothesis was to use one of the existing DHP. The main pitfall of DHP is the blockade of both Ca_V_1.2 and Ca_V_1.3 [139]. Hence, the selective pharmacological targeting of Ca_V_1.3 is not possible as both isoforms are concomitantly expressed in neurons. To complicate the issue, DHPs show a higher affinity for Ca_V_1.2 than Ca_V_1.3 [134,140]. Among DHPs, isradipine shows a high affinity for Ca_V_1.3, although preferential selectivity for Ca_V_1.2 persists [141]. Therefore, isradipine has been the DHP candidate of choice for the clinical trials on PD.

A Phase-II clinical trial demonstrated that 10 mg/day is the maximal daily dosage of isradipine tolerated by early PD patients who do not yet require dopaminergic therapy [142]. Considering its short half-life, isradipine was administrated twice a day, 5 mg for each dose. The most common side effects were peripheral edema and dizziness [142]. This therapeutic regime was then used for a thirty-six-month randomized Phase-III trial, to test the efficacy of isradipine in delaying the clinical progression of PD in early-diagnosed patients [143]. Participants were tested on their ability to score using the unified Parkinson’s disease rating scale (UPDRS)—including cognitive functions, daily living activities, and motor function, which are all sensitive to anti-Parkinson’s medications—the time to onset of severe motor complications and the initiation of standard anti-Parkinson’s therapy. Despite the researchers’ high hopes, treatment with isradipine failed to score positively against the placebo for all these endpoints. Thus, the results of the clinical trial did not support the hypothesis that isradipine, at this dosage, can slow the progression of PD [143]. One possible explanation for this result is that the bioavailability of isradipine at the used dosage was not sufficient to target the Ca_V_1.3 channels in neurons, but a direct empiric measure of effective local drug engagement is not feasible [143,144]. This explanation has been further supported by modeling the pharmacokinetics of isradipine based on the trial data, indicating that the critical threshold for therapeutic efficacy might have been reached only transiently and for a short time [144]. The administration of higher doses is discouraged because of the secondary cardiovascular effects that isradipine may induce. The most effective strategy by which to test the therapeutic efficacy of blocking Ca_V_1.3 in PD would be the identification of Ca_V_1.3-selective inhibitors. Ideally, such inhibitors should be able to target the Ca_V_1.3 channels in neurons and not in the other tissues where they are expressed, such as the cardiac sinoatrial node, endocrine system, and the cochlea.

Finally, R- and T-type VGCCs are emerging as possible therapeutic targets for PD [8]. For example, the compound NNC 55-0396 was shown to offset locomotor deficits in a rodent model of PD by inhibiting the T-type channels [145]. Furthermore, the activity of T-type VGCCs was recently found to mediate the dysregulation of calcium homeostasis in PARK6 patient-specific-induced pluripotent stem cells [146]. Therefore, the inhibitors of T- type channels could represent a valid strategy in PD treatment [147,148]. Recent advances and biomedical findings support this possibility and are extensively discussed in a recent review [149].

## 6. The Potential of Pyrimidine-2,4,6-Triones (PYT) as Ca_V_1.3 Selective Inhibitors

The clinical need for selective Ca_V_1.3 blockers does not apply only to PD. Indeed, gain-of-function mutations of Ca_V_1.3 are associated with autism and epilepsy [116,150]. In the ventral tegmental area, Ca_V_1.3 is involved in cocaine addiction and related comorbid mood disorders [151]. In addition, genetic data identify *CACNA1D* as being a risk factor for bipolar disorders [45,46]. The L-VGCCs inhibitors used in clinical practice, such as isradipine, verapamil, and diltiazem, show a higher affinity for Ca_V_1.2 rather than Ca_V_1.3 channels [140,141]. Consequently, we can expect that the significant inhibition of Ca_V_1.3 in the brain would require the administration of high doses of calcium channel antagonists, leading to cardiovascular side effects induced by the blockade of Ca_V_1.2. Therefore, the effective inhibition of Ca_V_1.3 in the central nervous system requires selective molecules sparing Ca_V_1.2. The interest of the scientific community is high, and several laboratories are currently testing innovative compounds targeting Ca_V_1.3. These compounds could be of great interest for both basic science and therapeutics. A novel class of small molecules, pyrimidine-2,4,6-triones (PYT), has been indicated as a potential molecular paradigm for generating possible Ca_V_1.3-selective inhibitors. In particular, 1-(3-chlorophenethyl)-3-cyclopentylpyrimidine-2,4,6-(1*H*,3*H*,5*H*)-trione) (also known as Compound 8 (or PYT06 in [152])) was shown to be highly selective for Ca_V_1.3 (IC_50_ = 24.3 ± 0.7 μM) over Ca_V_1.2 (1162 μM) [153]. The structural bases for its selectivity to Ca_V_1.3 and voltage-dependent inhibition mechanism of channel gating were recently identified [154]. Compound 8 binds to the Ca_V_1.3 α_1_ subunit in the DHP-binding pocket in a voltage-dependent way, which confers negative allosteric modulation [154]. However, electrophysiology recordings in HEK-293 cells expressing various combinations of Ca_V_1.3 or Ca_V_1.2 α_1_ splice variants with different Ca_V_β isoforms show that the selectivity of Compound 8 for Ca_V_1.3 is modest and is highly dependent on the molecular identity of the channel complex [155]. Intriguingly, Ortner et al. (2014) [156] showed that under their experimental conditions, Compound 8, rather than reducing L-type currents, increased calcium influx through Ca_V_1.3 and Ca_V_1.2 by slowing current activation and inactivation, as well as enhancing tail currents in HEK-293 cells expressing the channel subunits and in chromaffin cells. In the same study, the weak inhibition of L-type currents occurred only when using Ba^2+^ as a charge carrier, but no selective action on Ca_V_1.3 over Ca_V_1.2 was observed [156]. These discrepancies were in part explained by the presence of a critical mutation in the DHP-binding pocket—the interaction site of Compound 8—of the Ca_V_1.3 α_1_ subunit used by Ortner et al. (2014), which could impede the proper interaction of Compound 8 with the channel pore [154,156]. The enhancement of tail currents could be interpreted as the effect of a secondary binding site on the channel, which became evident in the absence of a higher-affinity binding on the DHP pocket [154,156]. However, the mutated DHP-binding site could not explain the agonist action of Compound 8 on native L-type currents in chromaffin cells, as reported by Ortner et al. [156]. Nonetheless, the inhibitory function of Compound 8 on L-Type currents was observed in neurons in another study [157]. While the mechanisms underlying the action of Compound 8 on L-type channels are controversial, these studies indicate that the cellular environment, subunit splice variants forming the channel complex, and the neuronal firing mode affect the action of Compound 8 on L-VGCCs. Further characterization in native cells expressing Ca_V_1.3—for example, different types of neurons, sinoatrial node myocytes, pancreatic beta cells, and chromaffin cells—will be necessary to understand the mechanism of action of Compound 8. The outcome of these investigations could provide important information on the tissue-specific effects of this molecule. These findings will be useful to develop pharmacological treatments for *CACNA1D*-dependent neuropsychiatric disorders and for the evaluation of potential side effects.

## 7. VGCCs Inhibitors in Pain Treatment

Pain stimuli are detected by peripheral nociceptors innervating the skin and organ tissues [9]. Then, action potentials propagate along the primary afferent fibers to the synapses in the spinal dorsal horn, where the excitatory synaptic transmission connects to those brain centers coding pain [8,158]. In dorsal horn neurons, Ca_V_3.2 VGCCs participate in nociceptive pathways by regulating membrane excitability, and, to a lesser extent, synaptic transmission. Conversely, Ca_V_2.2 is the main regulator of synaptic transmission [8,51]. Ca_V_2.2 and Ca_V_3.2 are upregulated in conditions of chronic pain [52,53,54], while their inhibition mediates analgesia in mice [51]. The inhibition of Ca_V_2.2 constitutes a prime pharmacological strategy to implement efficient pain therapy. Ca_V_2.2 are known to form complexes with µ-opioid receptors. The administration of the µ-opioid receptor agonist morphine inhibits Ca_V_2.2, reduces neurotransmitter release from primary afferent neurons, and exerts a powerful analgesic function [55,56]. The expression of the Ca_V_2.2 variant containing the exon 37a plays a central role in pain signaling [57,58]. Interestingly, an alternative splicing of Ca_V_2.2 at exons 37a and 37b diminishes the efficacy of morphine, probably by altering the composition of the Ca_V_2.2 complex with µ-opioid receptors, preventing channel regulation by morphine [55]. The same study showed that the analgesic efficacy of gabapentin and Ziconotide is not affected by Ca_V_2.2 alternative splicing [55]. Such difference is attributable to the different mechanisms of action of these drugs with respect to morphine. Ziconotide acts by occluding the channel pore, whereas gabapentin targets the Ca_V_α_2_δ subunits, inducing channel pore α_1_ internalization [55,59]. The Ca_V_α_2_δ subunits are upregulated in chronic pain states, determining an increase of Ca_V_2.2 trafficking and localization at synapses [60,61]. Gabapentinoids reduce the expression levels of Ca_V_2.2 at the presynaptic membrane by binding to Ca_V_α_2_δ. This hampers synaptic transmission, thereby reducing the efficacy of nociceptive signaling [159]. Ziconotide is delivered intrathecally to treat pain in cancer patients. It has several disadvantages, including its mode of administration—which depends on a minipump implant—and numerous and severe side effects [160,161,162,163]. These side effects are possibly due to the lack of state-dependence of the Ziconotide blockade of calcium channels. Indeed, Ziconotide would block channels irrespective of the basal or hyperactive firing of neurons, whereas a state-dependent inhibitor would preferentially target the channels in hyperactive neurons. Along the same lines, several use-dependent small molecules inhibiting Ca_V_2.2 that are also capable of analgesic activity on animal models were developed over the years. Some of these molecules, such as TROX-1, Z160 (also named NMED-160 or NP-118809), and CNV2197944 entered clinical trials [8,164,165].

Small molecules isolated from the rhizome and roots of *Valeriana jatamansi* Jones (Caprifoliaceae), an annual herb mainly found in China and India [166], show significant inhibition of Ca_V_2.2 and Ca_V_3.1 channels. These molecules exhibit selectivity for Ca_V_2.2 and Ca_V_3.1 against Ca_V_1.2, Ca_V_2.1, and KCNH2 [167]. Together with other blockers of Ca_V_2.2 endowed with analgesic properties in animal models [168,169], these molecules may represent an attractive option for exploring novel possibilities for treatment in pain therapy targeting VGCCs. Another possibility could be interfering with Ca_V_2.2 trafficking to the membrane so that the presynaptic amount of calcium channels would be reduced, and the transmission of nociceptive stimuli would be inhibited. A recent example of this strategy exploits hot-spots at the interface of Ca_V_α_1_- Ca_V_β interaction, constituted by three critical amino acids: Tyr-437, Trp-440, and Ile-441 on the Ca_V_α_1_ pore-forming subunit [170,171]. By the structure-based screening of commercial libraries, the BTT-3 small molecule was selected and used as a molecular paradigm to develop BTT-266 and BTT-369—compound 6 and 14 in [170], respectively—which reduced Ca_V_2.2 trafficking to the membrane and modulated channel voltage-dependence activation and steady-state inactivation [170]. In mice, these compounds relieve pain with different duration and efficacy. The use of these molecules may pave the way to treating other channelopathies with aberrant α_1_ trafficking and biophysical properties [170]. Similarly, small molecules mimicking the interaction of STAC3 with Ca_V_1.1 could offset the abnormal muscle physiology of Native American myopathy [172,173].

## 8. VGCCs in Seizure Disorders

Seizures originate from membrane hyperexcitability and/or the abnormal synchronization of neurons in the brain, which perturbs the physiological pattern of neuronal circuitry [8,174]. Proper connections between neural circuits permit the coordination of different tasks and behaviors. Thus, the disruption of normal interconnectivity may account for epilepsy comorbidities, such as depression, learning disabilities, and autistic features [174]. Within the epileptic focus, seizures are believed to derive from increased excitation or decreased inhibition and can be determined by a brain tumor or damage to brain structures [8,174,175]. Conversely, idiopathic seizures are triggered by systemic conditions, such as fever or hypoxia. Genetic conditions of ion channels and GABA receptors are also involved in seizures [147,176,177,178]. In the past two decades, important advances in our understanding of the physiopathological mechanisms underpinning seizures have led to an increase in the available antiepileptic drugs. Nevertheless, about one-third of patients are refractory to validated pharmacological and medical treatments, while others suffer severe side effects [174,179,180,181,182,183]. Therefore, there exists an urgent need to develop novel treatments that are able to contain the extent and frequency of seizure episodes in drug-resistant patients and to minimize the adverse effects [174,179,184].

Several lines of evidence show that T-type VGCCs are involved in absence seizures. The expression level of Ca_V_3.2 mRNA and T-type currents increase in the reticular nucleus of the thalamus in absence epilepsy rats from Strasbourg (GAERS), a model of absence epilepsy [67]. Increased thalamic T-type currents are attributable to a gain-of-function mutation in exon 24 of Ca_V_3.2, identified in GAERS. Interestingly, the gain-of-function phenotype depends on the alternative splicing of Ca_V_3.2 exon 25 [68]. Furthermore, mutations within the *CACNA1H* gene encoding Ca_V_3.2 have been associated with several forms of epilepsy [69]. These mutations generate gain-of-function channels or enhance channel trafficking to the neuronal membrane, thereby increasing the amount of functional surface that expressed Ca_V_3.2 [70,71,72]. Mice overexpressing Ca_V_3.1 channels show increased thalamocortical activity and absence seizures [66]. Recently, gain-of-function Ca_V_3.3 channel mutants, identified in patients with seizures and neurodevelopmental disorders, were shown to cause hyperexcitability when expressed in chromaffin cells; this finding could explain seizures in patients [74]. Overall, these data indicate that enhanced T-type currents in the thalamus predispose a sensitivity to absence seizures. Therefore, the inhibition of Ca_V_3 channels represents a valid strategy for the pharmacological treatment of seizures.

The T-type VGCCs blocker ethosuximide is used in the treatment of absence seizures [185]. This small molecule exerts its action on all Ca_V_3 isoforms and binds with a higher affinity to inactivated channels [186]. However, ethosuximide is rather unspecific as it was also shown to inhibit voltage-gated sodium channels and calcium-activated potassium channels in the thalamic and cortical neurons [187,188]. Moreover, ethosuximide administration increases GABA levels and decreases glutamate in GEARS [189]. Among the anti-epileptic drugs, sodium valproate can inhibit T-type currents in addition to sodium channels [190]. Zonisamide is used to control seizures and was also shown to inhibit T-type VGCCs. In addition, Zonisamide relieved pain responses in rodents, corroborating the role of these channels in the nociceptive pathways [191,192]. The experimental evidence for the involvement of T-type VGCCs in seizures prompted the development of a novel molecule based on the rational design of NP118809 (or Z160), a high-affinity N-type channel blocker able to control pain in animal models of inflammatory and neuropathic pain [164,193]. This approach led to Z944, a high-affinity pan-T-type blocker, exhibiting state- and frequency-dependent effects and that was able to reduce seizures by 85–90% in GAERS [193].

Gabapentin and pregabalin are used in clinical practice to treat focal and partial seizures [194]. Because these drugs bind to the Ca_V_α_2_δ subunit, they induce several unwanted effects by targeting multiple VGCCs, irrespective of the Ca_V_α_1_ isoform. Furthermore, in patients treated for neuropathic pain with drugs targeting VGCCs, such as benzodiazepines and opioids, the use of gabapentinoids is critical because of possible pharmacodynamic interactions [194].

The antiepileptics lamotrigine and topiramate target multiple channels and receptors, and both were shown to inhibit Ca_V_2.3 channels, among others [62,63]. Indeed, in rodents, the anti-seizure effect of lamotrigine is critically dependent on the expression of Ca_V_2.3, and it is lost in Ca_V_2.3-null mice [64]. Topiramate blocks Ca_V_2.3 in a state-dependent manner, meaning that mainly hyperactive neurons are targeted [65].

L-type Ca_V_1.2 channels were proposed to be involved in the onset of febrile seizures [195]. Indeed, the activation of Ca_V_1.2 in pyramidal neurons is shifted to hyperpolarized potentials at a temperature of about 40 °C, allowing these channels to support intrinsic firing properties and, therefore, likely supporting febrile seizures [195]. Consistently, nimodipine prevented the development of temperature-induced seizures in rodents, indicating that L-type channel blockers could be explored as a pharmacological tool to treat febrile seizures [195]. However, it is well known that nimodipine also slightly blocks T-type channels [196]. Therefore, the relative contributions of L-type and T-type channels in febrile seizure and in the protecting effect of nimodipine need to be clarified.

## 9. VGCCs in Migraine

Familial hemiplegic migraine 1 is caused by the S218L mutation of Ca_V_2.1, which alters the kinetic properties of the channel currents and hampers proper synaptic formation and synaptic plasticity [49,50]. A small molecule termed 2,5′-di(tertbutyl)-1,4,-benzohydroquinone (BHQ)—primarily known as the SERCA inhibitor—confers a dual effect on Ca_V_2.1 channels by inhibiting voltage-dependent activation and enhancing calcium-dependent facilitation [49]. The use of the BHQ on the Ca_V_2.1-S218L mutant rescues normal current properties and restores proper synaptic physiology in *Drosophila* and animal models [49]. These results show that reestablishing normal channel kinetics rescues the disease phenotype and indicates a strategy by which to treat familial hemiplegic migraines in humans.

## 10. VGCCs in the Aging Brain

VGCCs undergo age- and gender-dependent alternative splicing, suggesting that different ratios of precise splice variants may support changes in the aging brain [197]. Age-dependent forms of mid-channel proteolysis, with the generation of Ca_V_1.2 with diverse biophysical properties, were reported [198]. Mid-channel proteolysis may serve as a homeostatic control of VGCCs activity. This hypothesis is supported by the finding that proteolysis can be reduced by inhibiting L-VGCCs with the DHP nifedipine in cultured neurons and slices [198]. In aging mice, neuronal Ca_V_1.2 exhibited higher levels of phosphorylation on serine 1928, which increases open-channel probability [199,200]. Ca_V_1.2 phosphorylation may also be involved in the regulation of channel trafficking in the hippocampal neurons [201]. Thus, knowing the age-dependent regulation of VGCCs might offer therapeutic strategies to compensate for the consequences of changes in neuronal calcium homeostasis that are typical of later life.

## 11. Summary

VGCCs are involved in several neurological and psychiatric conditions. However, the palette of molecules targeting these channels is limited, applying only to some channel subtypes, and is restricted to an inhibitory function. There exists the need to identify novel specific modulators and inhibitors that could be considered for use in clinical practice. The topics discussed in the previous paragraphs highlight two main points. First, compounds in use for some disease may be considered also for other conditions (Table 2). Second, several issues could be exploited in evaluating new small molecules toward VGCC-dependent pathologies. These aspects include an understanding of the tissue- and function-specific channel biophysical properties, splice variant expression patterns, and the molecular composition of signaling complexes and transduction cascades. Based on these notions, the researcher can direct drug development toward the most effective strategies.

Current research is progressively integrating those findings provided by genetic screenings with the molecular and cellular mechanisms downstream of calcium channels that are involved in diseases. Together with structural data on channel complexes, these notions are crucial for screening existing small molecule libraries or planning the rational design of substances already in use. Ameliorating the clinical course of VGCC-dependent diseases still requires considerable transdisciplinary research efforts.

## Figures and Tables

**Table 1:** Subtype, function, and disease of calcium channel types.

Current Type	Ca_V_ Nomenclature	Specific Blocker	Gene	Main Physiological Role	Disease
L	Ca_V_1.1	DHP	*CACNA1S*	Excitation-contraction coupling in skeletal muscle, regulation of gene transcription	Hypokalemic periodic paralysis [5], normokalemic periodic paralysis; malignant hypothermia susceptibility [5]
	Ca_V_1.2	DHP	*CACNA1C*	Excitation-contraction coupling in cardiac muscle, regulation of gene transcription, endocrine secretion, spine and dendritic calcium signaling in neurons	Timothy syndrome [25,26,27,28,29], bipolar disorder [30,31], depressive disorder [32,33,34], schizophrenia [33,35,36,37,38,39], post-traumatic stress syndrome [40,41], Brugada syndrome (^#^ 611875), cardiac Long QT syndrome [^#^ 618447]
	Ca_V_1.3	DHP	*CACNA1D*	Hearing, cardiac and neuronal pace-making activity, spine and dendritic calcium signaling in neurons	Deafness [42,43], autism [44], bipolar disorder [45,46], sinoatrial dysfunction (^#^ 614896)
	Ca_V_1.4	DHP	*CACNA1F*	Retinal neurotransmission	Congenital stationary night blindness [47,48], X-linked Cone-Rode dystrophy (^#^ 300476), Aland Island eye disease (^#^ 300600)
N	Ca_V_2.1	ω-conotoxin-GVIA	*CACNA1A*	Neurotransmitter release, somatodendritic calcium signaling	Familial hemiplegic migraine [49,50], ataxia (^#^ 108500, ^#^ 183086)
P/Q	Ca_V_2.2	ω-agatoxin-IVA	*CACNA1B*		Pain [8,51,52,53,54,55,56,57,58,59,60,61], neurodevelopmental disorder ^#^ 618497
R	Ca_V_2.3	SNX-482	*CACNA1E*	Neurotransmitter release, membrane excitability	Seizure [62,63,64,65], neurodevelopmental disorder(^#^ 618497), encephalopathy (# 618285)
T	Ca_V_3.1	Ethosuximide Zonisamide	*CACNA1G*	Membrane excitability, pace-making, firing, subthreshold oscillations	Seizure [66], spinocerebellar ataxia (^#^ 616795 and ^#^ 618087)
	Ca_V_3.2	Ethosuximide Zonisamide	*CACNA1H*		Seizure [67,68,69,70,71,72], autism [73], pain [51,52,53,54], hyperaldosteronism (^#^ 617027)
	Ca_V_3.3	Ethosuximide Zonisamide	*CACNA1I*		Seizure and neurodevelopmental disorders [74]

Note: ^#^ indicates the reference number in the “Online Mendelian Inheritance in Man” (OMIM) database for channelopathies.

**Table 2:** The applications of selected VGCC blockers and modulators in neurological and psychiatric conditions.

Small Molecules	Approved Applications	Target	Potential Applications ^#^
Isradipine	Hypertension	L-type channels	Autism [44,116], failed Phase-III trial for PD [116], dependency [151]
Nimpodipine	Hypertension	L-type channels	Anxiety [121], febrile seizures [195]
Roscovitine	NA	Ca_V_1.2, L-type currents	Timothy syndrome [29,114]
Pregabalin	Pain and seizures	Ca_V_α_2_δ	Anxiety [119]
Gabapentin	Pain and seizures	Ca_V_α_2_δ	Anxiety [119]
NNC 55-0396	NA	T-type currents	PD [145]
Valeriana jatamansi derived small molecules	NA	Ca_V_2.2, Ca_V_3.1	Pain [167]
Ziconotide	Pain	Ca_V_2.2	NA
BTT-266, BTT-369	NA	β binding domain on α_1_	Pain [170]
Ethosuximide	Seizures	T-type channels	Pain [185]
Valproate	Seizures	T-type channels	PD [190,191]
Zonisamide	Seizures	T-type channels	Pain and PD [191,192]
NP118809 (or Z160)	NA	N-type channels	Pain [164,193]
Z944	NA	T-type channels	Seizures, pain [193]
Lamotrigine	Seizures	R-type channels	Pain [62]
Benzohydroquinone	NA	Ca_V_2.1	Familial hemiplegic migraine 1 [49]

NA, not applicable; ^#^ potential applications are given on the basis of preclinical findings.

## Data Availability

Not applicable.
